# Insights into Mechanical Behavior and Biological Properties of Chia Seed Mucilage Hydrogels

**DOI:** 10.3390/gels7020047

**Published:** 2021-04-20

**Authors:** Pasquale Sacco, Sara Lipari, Michela Cok, Matilde Colella, Eleonora Marsich, Francesco Lopez, Ivan Donati

**Affiliations:** 1 Department of Life Sciences, University of Trieste, Via Licio Giorgieri 5, 34127 Trieste, Italy; sara.lipari97@gmail.com (S.L.); mcok@units.it (M.C.); idonati@units.it (I.D.); 2 Department of Biosciences, Biotechnology and Biopharmaceutics, University of Bari “Aldo Moro”, Via Orabona, 4, 70126 Bari, Italy; matilde.colella@uniba.it; 3 Department of Medicine, Surgery and Health Sciences, University of Trieste, Piazza dell’Ospitale 1, 34129 Trieste, Italy; emarsich@units.it; 4 Department of Agricultural, Environmental and Food Sciences (DiAAA) and Center for Colloid and Surface Science (CSGI), University of Molise, Via De Sanctis, 86100 Campobasso, Italy; lopez@unimol.it

**Keywords:** chia seeds, mucilage, hydrogels, mechanical behavior, cytotoxicity, osteogenesis

## Abstract

In this contribution we report insights on the rheological properties of chia (*Salvia hispanica*) seed mucilage hydrogels. Creep experiments performed in steady state conditions allowed calculation of Newtonian viscosities for chia hydrogels with different polymer concentration, pointing at inter-chain interactions as the main responsible for the different behavior toward network slipping under constant stress. A combination of oscillatory frequency and stress sweep tests highlighted a moderate effect of temperature in influencing hydrogel mechanics. The latter results prompted us to investigate potential biological functions for this set of biomaterials. Lactate Dehydrogenase assay proved the lack of cytotoxicity of chia suspensions toward Human Mesenchymal Stem Cells from adipose tissue used here as a cell model. Differentiation experiments were finally undertaken to verify the influence of chia samples on osteo-induction triggered by chemical differentiation factors. Alkaline Phosphatase enzyme activity assay and Alizarin red staining demonstrated that chia mucilage did not alter in vitro stem cell differentiation. Collectively, this set of experiments revealed an almost inert role associated with chia suspensions, indicating a possible application of chia-based networks as scaffold models to study osteogenesis in vitro.

## 1. Introduction

Hydrogels are known as water-swollen cross-linked polymers characterized by covalent bonds or physical interactions, endowing such matrices with peculiar mechanical properties. The nature of molecular interactions is of pivotal importance to modulate their final application [1,2,3,4,5,6]. Hydrocolloids, for example, find application in several fields ranging from food to biomedical technologies [1,3,7]. Hydrocolloids are frequently used in food industry as gelling agents or thickeners since they are able to retain a discrete amount of water [8] and to modify the food structure. Interestingly, the hydrocolloids used in food industry include natural polymers that offer a versatile degree of flexibility for many tasks: sodium alginate, agar, carrageenan, locust bean gum, guar gum, gum arabic, xanthan gum and gellan gum are used in food preparations as additives [9,10,11].

Samateh and colleagues demonstrated that hydrogel-like structures can be obtained in the absence of external cross-linkers by some seeds like those of chia (*Salvia hispanica*) and basil (*Ocimum basilicum*). When soaked in water these seeds form a hydrogel-like matrix characterized by an extensive network of nanoscale fibers protracted from the seeds’ surface into the aqueous bulk. The set of secreted fibers from the seeds is indicated in various studies as mucilage [12].

*Salvia hispanica* is an annual herbaceous crop species of the Lamiaceae family, native to Mexico and Guatemala [13]. Currently, the nutritional benefits of chia seeds have been validated and their effects on human health documented [1,14,15]. Due to the high content of ω-3 and ω-6 fatty acids, vitamins, antioxidants and minerals, chia seeds constitute a widely used ingredient in food industry applications [16]. Chia seeds, besides being a source of oil and protein, are certainly a source of polysaccharides [17], and for this reason they represent suitable candidates for the fabrication of interesting materials like emulsion systems in food industry. Munoz and colleagues [18] studied the mucilage release from the seed coat during hydration, finding that it can be easily extracted and hydrated to achieve water retention worth 27-fold its weight, thus underlying the great potential of chia seed mucilage as a functional ingredient to be used as a thickener in foods. Since then, the interest toward the chia seed mucilage has increased greatly in the literature [19,20,21,22].

Salgado-Cruz et al. [23] characterized the chia seeds and the mucilage produced from the seeds when soaked in water. The authors, through microscopic imaging, observed the seed hydration process and the release of the mucilage. The specific composition of the chia mucilage has been reported by Coorey et al. [24] who determined in the freeze-dried hydrogel foam the contents of moisture, ash, protein, fiber, oil, and fatty acid profile. In particular, they found a 58% content of crude fibers (insoluble fraction) and 35% of carbohydrates. They investigated the effect of pH, ionic strength and the type of cation in chia hydrogel properties. Though much work has been done over the last few years to investigate the structural characteristics of chia hydrogels, fundamental knowledge—especially referring to mechanical properties—still needs to be gained to shed more light on this interesting material. In fact, for its rheological characteristics chia mucilage has been proposed as a fat replacer in different food preparations, like bread and cakes [25] and in dressing sauces [26]. Moreover, some attempts have been made for the production of edible films based on chia mucilage together with chia seed proteins and clove oil [22], and together with glycerol [27]. Recently, some of the authors proposed that chia polymer dissolved in water, in its gel state, is susceptible to autoxidation phenomena that can be avoided in the presence of an essential oil [28,29]. The importance of the structure–property relationship of polymer networks specifically related to the reduction in network defects, such as inhomogeneity, is well recognized [30]. This inhomogeneity typically originates in an unpredictable manner and can affect the resulting physical properties of the network.

In our previous investigation, by means of steady-shear flow tests on chia mucilage suspensions with different concentration, we showed that such suspensions can be classified as plastic fluids due the deviation from the Newtonian fluids behavior [29]. In the same study a decrease in the consistency index values (*k*) on temperature uprise was described, indicating that chia suspension (in the range of 0.2–1%) viscosity decreased with *k*, in line with the classic behavior of polymers in aqueous solutions [31].

Being a rich source of ω-3 (α-linoleic acid) fatty acids, chia seeds have been used in diets due to their beneficial influence on human health. In addition to the negligible cytotoxic effect on human preadipocytes and macrophages, chia seed extract has promising therapeutic applications. Thus, chia seed inhibits lipid accumulation and macrophage recruitment, thereby suppressing the inflammation associated with obesity [32].

In the last few years, the efforts of many researchers have been focused on the field of bone regeneration. The common trait of the numerous strategies under study is the design of osteochondral scaffolds that can mimic the structure, mechanobiological and physical–chemical functions of the bone tissue to allow proliferation and differentiation of stem cells [33]. In this research context, multipotent mesenchymal stem cells (MSCs) have been broadly used in both in vitro and in vivo models as well in clinical studies [34]. A fundamental requirement for proliferation and differentiation of stem cells into appropriate and functional tissue-specific cell types is an effective cell adhesion to biomaterials. Thus, a crucial role has been demonstrated to be played by the chemical composition, surface and biomechanical features of the biomaterials employed [35]. As such, the choice and the design of novel biomaterials that can mimic the structure, the mechanobiological and physical–chemical functions of the normal bone is crucial. Herbal extracts and mucilages obtained from fruit seeds are being broadly investigated in biomedical studies and applications for their biocompatibility, biodegradability, antioxidant and antibacterial properties. Interestingly, Quince seed mucilage-based scaffolds have recently been suggested as a novel alternative for natural polymers in skin tissue regeneration approaches [36].

In this work, on the basis of our previous data on the mechano-properties of chia seed mucilage, we further explored the mechanical characteristics of this hydrogel system and for the first time explored the suitability of this biomaterial for stem cell growth and differentiation. Thus, here we aim at building upon current knowledge about mechanical properties of chia hydrogels. Specifically, we focus on the role played by temperature and polymer concentration in influencing the viscoelastic nature of chia seed hydrogels. Furthermore, we propose a preliminary biological investigation on a human stem cell model to acquire information on the safety and differentiation properties of chia suspensions, a fundamental step toward the use of this biomaterial in the biomedical sector, especially for tissue engineering applications.

## 2. Results and Discussion

### 2.1. Mechanical Behavior

**Figure 1:**
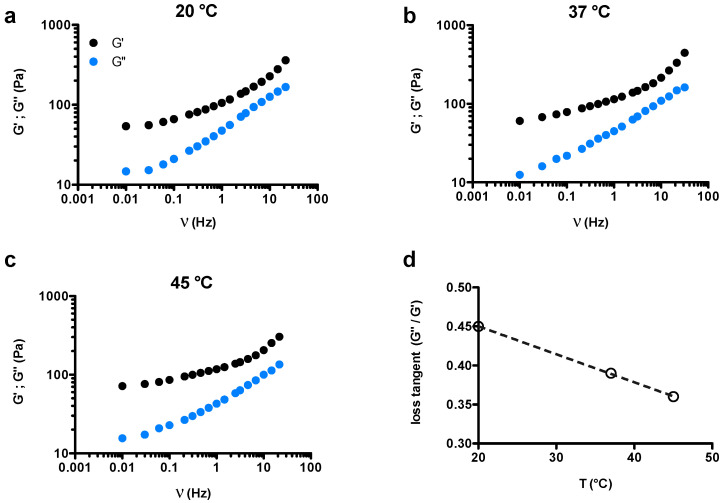
Frequency sweep investigation on chia hydrogels. (**a**–**c**) Hydrogels’ mechanical spectra recorded at different temperatures as a function of frequency, ν. (**d**) Dependence of loss tangent (tanδ=G″G′) calculated at 1 Hz on temperature. Experimental conditions: (chia) = 2% *w*/*w*, deionized water as solvent.

**Figure 2:**
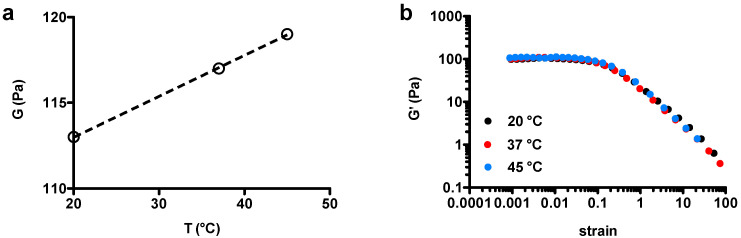
(**a**) Dependence of chia hydrogels’ shear modulus, *G*, on the temperature; the dashed line is drawn to guide the eye. (**b**) Dependence of the elastic modulus, $G^{\prime }$, on total strain applied at the different temperatures tested. Experimental conditions: (chia) = 2% *w*/*w*, deionized water as solvent.

First, mechanical analysis was carried out on 2% *w*/*w* chia hydrogels. Freeze-dried chia dissolved in water, after a visual inspection, revealed a significant turbidity enhancement, as well as important viscosity increase [29]. As reported in Figure 1, chia samples behaved as viscoelastic materials as demonstrated by the frequency-dependence of both elastic and viscous moduli. Furthermore, they appeared as (weak) gel-like networks at the three temperatures investigated, as demonstrated by larger G′ values than G′′ across a range of frequencies spanning almost three orders of magnitude. Loss tangent (=G″G′) at 1 Hz was considered as the parameter to study the effect of temperature on gel-like magnitude. We detected a weak linear decrement in loss tangent as a function of temperature, clearly indicating that chia samples mildly stiffened upon increasing the temperature (Figure 1d). This observation was confirmed by the trend of shear modulus calculated in the linear stress–strain regime as a function of temperature (Figure 2a). Present results differ from what found in the low chia concentration regime (0.2–1% *w*/*w*) [28], where a decrease in the consistency index (*k*) linked to the decrement of viscosity on temperature uprise was described.

The extent of linear viscoelastic regime was next evaluated by performing stress sweep experiments on chia hydrogels at different temperatures (Figure 2b). All samples analyzed manifested strain softening behavior at large deformations, with no apparent influence of the temperatures tested, the magnitude of the phenomenon being comparable among the chia suspension samples analyzed. The critical deformation value ($\gamma_{c}$) at which strain softening manifests was then determined according to the Soskey–Winter model (Equation (1)) [37]

(1) $$G^{\prime }=G{{}^{\prime }}_{0}\frac{1}{1+{\left(b\gamma \right)}^{n}}$$

where *G′*_0_ is the limiting value of the storage modulus for $\gamma_{\to 0}$, while *b* and *n* are adjustable parameters. $\gamma_{c}$ indicates the limit of the linear regime and was defined as $\gamma_{c}=\frac{G^{\prime }}{{G^{\prime }}_{0}}=0.95$ [38]. Again, non significant differences were detected among the samples analyzed, being $\gamma_{c}\;\sim \;1.5\%$ in all the chia suspensions analyzed. As a whole, these experiments indicate that the temperature has a marginal role in extending the linear stress–strain region.

**Figure 3:**
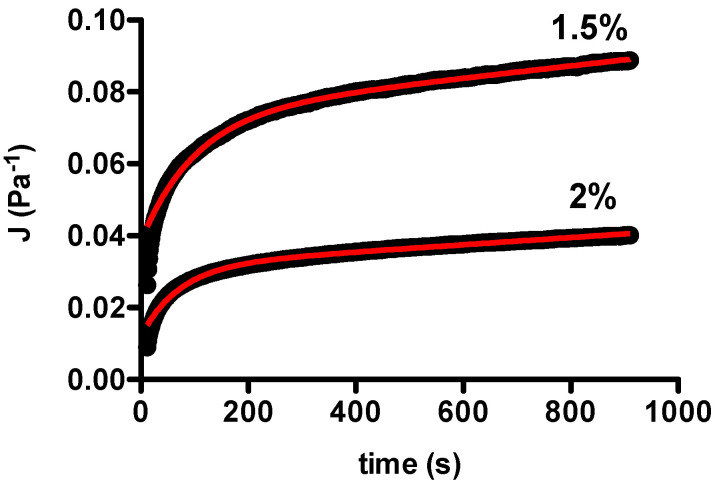
Dependence of creep compliance, *J*, on time for chia hydrogels with different polymer concentrations; solid red line represents the best fit of experimental points according to the Equation (2) reported in the main manuscript. Experimental conditions: (chia) = 1.5 or 2% *w*/*w*, deionized water as solvent.

The viscoelastic properties of chia hydrogels with different concentrations, namely 1.5 and 2% *w*/*w*, were next investigated in steady state conditions by creep experiments (Figure 3). To this aim, the two samples were subjected to an initial constant stress—well within linear viscoelastic regime—and the time-dependent compliance (J=τγ) was recorded as a function of time.

The creep compliance curve profiles were analyzed by means of a model composed of a Maxwell element in series with one Voigt element (Equation (2)), which accurately fitted the experimental data [39]

(2) J(t)=J0+J1(1−e−tλ)+tηN

where J(t) is the measured compliance, $J_{0}$ and $J_{1}$ are the compliances of the Maxwell and Voigt springs, respectively, $\eta_{N}$ is the so-called Newtonian viscosity of the Maxwell dashpot, and λ is the retardation time associated with the Voigt element. The results obtained for the two samples are reported in Table 1. Though similar values of both $J_{0}$ and $J_{1}$ were obtained, solid speculations can be drawn on the basis of Newtonian viscosities of chia samples with different concentrations. Since $\eta_{N}$ is affected by interchain interactions—and hence polymer concentration or crosslinking density—2% chia hydrogels endured better toward network slipping than their 1.5% counterparts. 

### 2.2. Biological Properties

**Figure 4:**
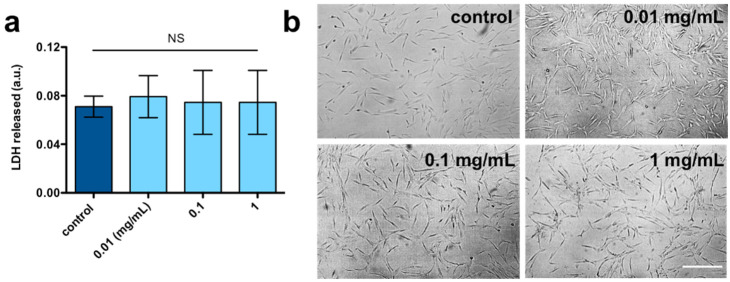
(**a**) Lactate Dehydrogenase enzyme released from hMSC-AT cells when incubated with chia suspensions with different concentrations. Data are reported as mean ± s.d., *n* = 3–4 samples analyzed for each experimental condition. Statistics: NS, not significant (one-way ANOVA followed by Dunnett’s Multiple Comparison post hoc test). (**b**) Optical microscopy of hMSC-AT cells in the presence or not of chia suspensions with different concentrations (range 0.01–1 mg/mL). Images were acquired with a digital camera (scale bar is 200 μm).

To evaluate the safety of chia suspensions for potential uses in biomedical or food applications, an LDH assay was carried out on a Human Mesenchymal Stem Cell model (hMSC-AT cells). Adherent cells were incubated with different concentrations of chia suspensions for 24 h, and the amount of LDH enzyme quantified colorimetrically (Figure 4a). LDH levels were almost comparable between chia-treated cells with respect to control cells, indicating that chia suspensions can be safely classified as non-hazardous polymer materials. Optical microscopy confirmed the lack of cytotoxicity associated with chia suspensions, with hMSC-AT cells exhibiting a regular morphology (Figure 4b).

**Figure 5:**
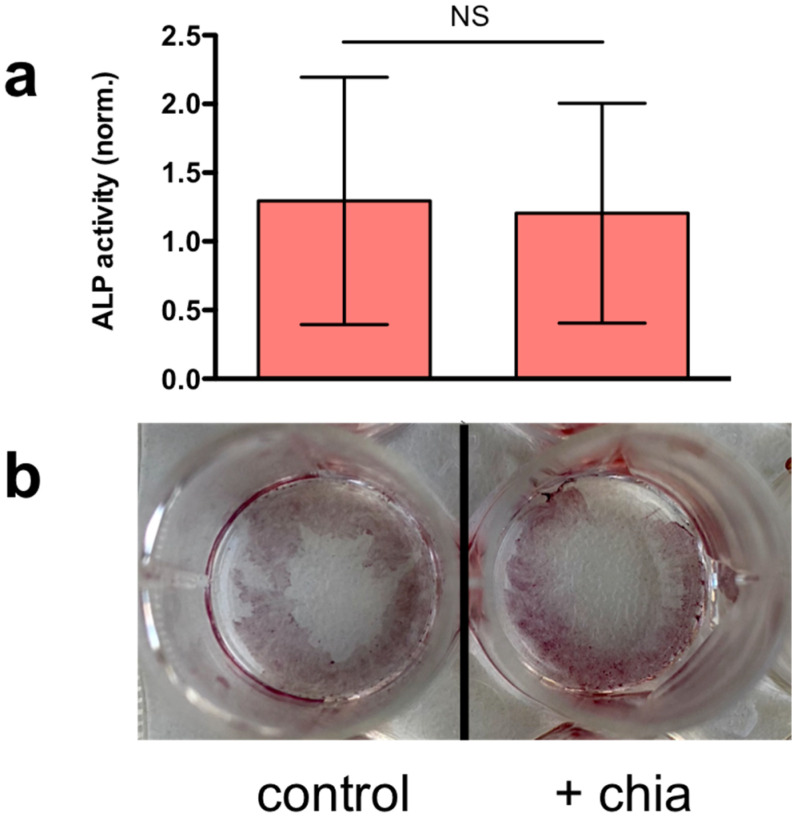
(**a**) Quantification of alkaline phosphatase (ALP) activity of hMSC-AT cells when incubated for 14 days with chia suspension (1 mg/mL) dissolved in differentiation medium. Data are reported as the mean ± s.d., *n* = 6 samples analyzed for each experimental condition. Statistics: NS, not significant (two-tailed Student’s *t*-test). (**b**) Representative images of calcium deposits from control and chia-treated hMSC-AT cells after 14 days of differentiation.

Next, we investigated whether chia suspensions can modulate or interfere with hMSC-AT in vitro differentiation towards a bone-like phenotype. We assessed the osteogenic differentiation through classical assays [40]: a quantitative assay of alkaline phosphatase (ALP) activity upon cell lysis and a qualitative Alizarin Red staining to detect calcium deposits [35,41,42] (Figure 5). Collectively, we did not find significant differences between chia-treated cells and the control group after 14 days of incubation in differentiation medium in both the assays, meaning that chia did not interfere with osteogenic differentiation guided by the differentiation factors included in the cell culture medium.

## 3. Conclusions

As a whole, the data collected contribute to building upon current knowledge about mechanical properties of chia hydrogels. More in detail, the results show a weak dependence of viscoelastic properties of chia hydrogels as a function of temperature, with mild stiffening detected upon increasing temperature. Furthermore, chia hydrogels manifested strain softening behavior at large deformations. Creep experiments were useful in giving insights into viscoelastic behavior of chia hydrogels under steady state conditions. Indeed, calculation of Newtonian viscosities for chia hydrogels with different polymer concentrations highlighted that the whole polymer network is affected by inter-chain interactions, which are responsible for a different behavior toward network slipping under constant stress applied. Taken together, our findings provide additional information for expanding the current understanding on this intriguing polymer material from the mechanical side. Finally, here we report biological data about in vitro safety of chia suspensions toward human stem cells. Of note, we have preliminary demonstrated that chia suspensions do not interfere with the osteogenic differentiation of this stem cell line upon stimulation with differentiation factors, indicating a possible application of chia-based networks as a scaffold model to study osteogenesis in vitro.

## 4. Materials and Methods

### 4.1. Materials

Chia seeds (Salba grain organic) were obtained from I.P.A. s.r.l. Industria Prodotti Agroalimentari (Viterbo, Italy). L-ascorbic acid, β-glycerophosphate, dexamethasone, Phosphate Buffer Saline (PBS), Tris-HCl, Triton X-100, magnesium chloride (MgCl_2_), para-nitrophenylsphoshate, Bradford reagent and Alizarin Red S were all from Sigma. Lactate Dehydrogenase Activity Assay Kit was from Abcam. DMEM (Dulbecco’s Modified Eagle’s Medium) High Glucose, Fetal Bovine Serum (FBS), streptomycin, penicillin and trypsin were bought from EuroClone (Milan, Italy).

### 4.2. Mucilage Extraction from Chia Seeds

Chia seeds were weighed and placed in a vessel with ultrapure water in a 1:20 (*w*/*v*) ratio for 12 h to extract the mucilage (CM). The CM was recovered through vacuum filtration with a Buchner funnel, frozen at −40 °C and then freeze-dried under a vacuum in a freeze-drier Genesis 25ES (VirTis, Stone Ridge, NY, USA) for 48 h (maximum shelves’ temperature +20 °C). After freeze-drying, CM, with the aspect of a dried foam, was stored at room temperature.

### 4.3. Hydrogel Preparation

CM hydrogels were prepared with different concentrations (1.5 or 2% *w*/*w*). The freeze-dried CM was dispersed in warm ultrapure water (*T* = 50 °C) under stirring until complete disappearance of macroscopic aggregates. CM hydrogels were analyzed immediately after the preparation. In the case of biological studies, CM hydrogels were prepared in Phosphate Buffered Saline buffer, sterilized by autoclaving and diluted to desired concentrations in cell culture medium (see below for further details).

### 4.4. Mechanical Analysis

Rheological measurements were performed using an HAAKE MARS III rheometer (Thermo Scientific, Waltham, MA, USA) operating in oscillatory and steady shear conditions. Cone-plate CP60/1° geometry was used to characterize CM hydrogels. Mineral oil was used to seal the interface between the two plates in order to improve thermal control and limit solvent evaporation. Mechanical spectra were recorded at various temperatures, namely *T* = 20, 37 and 45 °C, under oscillatory shear conditions, with constant applied stress, *τ*, of 1 Pa and frequency range 0.01–100 Hz. At the end of frequency sweep measurements, the extension of linear viscoelastic regime was determined through stress sweep experiments with *ν* = 1 Hz and stress range 1 < *τ* < 1000 Pa. Creep measurements were recorded in steady state conditions by following the evolution of total deformation as a function of time, with constant applied stress of 0.5 Pa.

### 4.5. Cell Culture Preparation for In Vitro Tests

Human Mesenchymal Stem Cells from adipose tissue (hMSC-AT, PromoCell, Heidelberg, Germany) were used for the in vitro experiments. Cells were cultured in DMEM (Dulbecco’s Modified Eagle’s Medium) High Glucose supplemented with 0.584 g/L L-glutamine, 0.11 g/L sodium pyruvate, 10% heat-inactivated fetal bovine serum (EuroClone, Milano, Italy), 1% penicillin/streptomycin (EuroClone, Italy), in a humidified atmosphere of 5% CO_2_ at *T* = 37 °C. In all experiments 30,000 cells/well were plated on 24-well plates. The day after seeding, cell culture medium was discarded and cells were treated or not treated with chia suspensions with different concentrations (1, 0.1 and 0.01 mg/mL, DMEM:PBS 90:10 *v*/*v* as medium). For the differentiation experiments, the medium was supplemented with 50 μg/mL L-ascorbic acid, 10 mM β-glycerophosphate and 0.1 μM dexamethasone (differentiation medium) [43]. The medium was changed every 3–4 days and experiments were performed after 14 days of differentiation.

### 4.6. Evaluation of Cytotoxicity: Lactate Dehydrogenase (LDH) Assay

In vitro cytotoxicity of CM suspensions toward hMSC-AT was evaluated by assessing the release of the LDH enzyme into the culture medium from the cytoplasmic compartment. The assay was performed according to the manufacturer’s instructions.

### 4.7. Analysis of Stem Cell Differentiation

To evaluate stem cell differentiation toward bone-like phenotype, Alkaline Phosphatase (ALP) enzyme activity was quantified [43]. Cells were retrieved after 14 days of incubation in the differentiation medium. After trypsinization (10 min), cells were collected into 1.5 mL Eppendorf tubes, rinsed with PBS (1 mL) twice and lysed for 30 min at −80 °C by adding 100 µL of *Lysis Buffer* (100 mM Tris-HCl, 0.2% *v*/*v* Triton X-100, pH 9.8). The supernatants (lysate) were then collected by centrifugation (12,000× *g* for 5 min) and 40 µL of resulting lysate were mixed with 40 µL of *Reaction Buffer* (100 mM Tris-HCl, 1 mM MgCl_2_, 6 mM paranitrophenylsphoshate, pH 9.8) into 96-well plates and incubated for 60 min at 37 °C under dark conditions. The reaction was stopped by adding 2 μL of NaOH (5 M) and the absorbance of samples was measured at *λ* = 420 nm using a FLUOStar Omega-BMG Labtech spectrofluorometer. Untreated cells were used as negative controls. Total protein concentration of lysate was determined through the Bradford assay using bovine serum albumin (BSA) to create a calibration curve (range 0.1–1.4 mg/mL). 5 µL of protein standards or samples were added to 250 µL of the Bradford Reagent. After incubation at room temperature, the absorbance was measured at *λ* = 590 nm. ALP activity was finally normalized for total protein concentration.

### 4.8. Alizarin Red S Staining

Detection of calcium deposits was determined using Alizarin Red S staining [41]. After 14 days of culture in differentiation medium, cells were fixed by adding paraformaldehyde 10% *v*/*v* (1 mL/well) and incubating at room temperature for 15 min. After extensive washing, samples were incubated with 2% of alizarin red solution (pH 4.2, Sigma-Aldrich, St. Louis, MO, USA) for 20 min at room temperature. After staining, samples were washed with deionized water to remove the excess of dye and 1 mL of deionized water was added to each well to acquire images by means of a digital camera.

## Figures and Tables

**Table 1:** Results from the analysis of the creep compliance curves through Equation (2).

Chia (%)	$J_{0}\;({\mathrm{Pa}}^{-1})$	$J_{1}\;({\mathrm{Pa}}^{-1})$	λ (s)	$\eta_{N}\;(\times 10^{5}\;\mathrm{Pa}\cdot s)$
1.5	0.04	0.04	101	0.6
2	0.01	0.02	71	1

## Data Availability

Not applicable.
